# Gated Organonanoclays for Large Biomolecules: Controlled Release Triggered by Surfactant Stimulus

**DOI:** 10.3390/nano12152694

**Published:** 2022-08-05

**Authors:** Elisa Poyatos-Racionero, Édgar Pérez-Esteve, Serena Medaglia, Elena Aznar, José M. Barat, Ramón Martínez-Máñez, Maria Dolores Marcos, Andrea Bernardos

**Affiliations:** 1 Instituto Interuniversitario de Investigación de Reconocimiento Molecular y Desarrollo Tecnológico (IDM), Universitat Politècnica de València, Universitat de València, Camino de Vera s/n, 46022 Valencia, Spain; 2 CIBER de Bioingeniería, Biomateriales y Nanomedicina (CIBER-BBN), Instituto de Salud Carlos III, 28029 Madrid, Spain; 3 Departamento de Tecnología de Alimentos, Universitat Politècnica de València, Camino de Vera s/n, 46022 Valencia, Spain; 4 Unidad Mixta de Investigación en Nanomedicina y Sensores, Instituto de Investigación Sanitaria La Fe, Universitat Politècnica de València, 46026 Valencia, Spain; 5 Unidad Mixta UPV-CIPF de Investigación en Mecanismos de Enfermedades y Nanomedicina, Centro de Investigación Príncipe Felipe, Universitat Politècnica de València, 46012 Valencia, Spain; 6 Departamento de Química, Universitat Politècnica de València, Camino de Vera s/n, 46022 Valencia, Spain

**Keywords:** organonanoclay, hematin, cyanocobalamin, oleic acid, surfactant, controlled release

## Abstract

The low toxicity and high adsorption capacities of clay minerals make them attractive for controlled delivery applications. However, the number of controlled-release studies in the literature using clay minerals is still scarce. In this work, three different clays from the smectite group (Kunipia F, montmorillonite; Sumecton SA, saponite; and Sumecton SWN, hectorite) were successfully loaded with rhodamine B dye and functionalized with oleic acid as a gatekeeper to produce organonanoclays for active and controlled payload-release. Moreover, hematin and cyanocobalamin have also been encapsulated in hectorite gated clay. These organonanoclays were able to confine the entrapped cargos in an aqueous environment, and effectively release them in the presence of surfactants (as bile salts). A controlled delivery of 49 ± 6 μg hematin/mg solid and 32.7 ± 1.5 μg cyanocobalamin/mg solid was reached. The cargo release profiles of all of the organonanoclays were adjusted to three different release-kinetic models, demonstrating the Korsmeyer–Peppas model with release dependence on (i) the organic–inorganic hybrid system, and (ii) the nature of loaded molecules and their interaction with the support. Furthermore, in vitro cell viability assays were carried out with Caco-2 cells, demonstrating that the organonanoclays are well tolerated by cells at particle concentrations of ca. 50 μg/mL.

## 1. Introduction

The need for numerous biomolecules to be protected and administrated in a sustained-and-controlled mode has been currently established as a major research area, especially in biomedicine. These requirements are principally due to two main reasons: to avoid biomolecule degradation, and to achieve an efficient absorption at the site, while unwanted side effects are minimized [1]. Mesoporous materials, and other structures that are able to be loaded, have become an interesting option for this purpose [2], with numerous examples, not only for biomedicine or pharmacology [3,4,5,6], but also for nutraceutical and food applications [7,8], or even for sensing and communication protocols [9,10,11,12,13].

Among the different types of loadable materials [10,14,15,16,17], clays (hydrated phyllosilicates) have a high potential for biological uses, among others, due to their inherent structure [18]. Clays are formed by compiled silica-and-alumina/magnesia layers, many of them being attached by exchangeable cations. Among all existing clays, minerals from the smectite group (as montmorillonite, hectorite or saponite) have a potential use in food, pharmaceuticals or nutraceuticals [19]. Smectite clays have especially high loading possibilities due to their high cation-exchange capacity compared with other clay minerals [20]. In addition, clays have a very low oral toxicity, so they have mild side effects derived from their intake [21]. Due to these characteristics, smectite minerals have been traditionally widely employed as excipients, and more recently as carriers for vitamins, drugs or proteins [22,23,24,25,26]. The literature shows that incorporation of these molecules into clay layers can result in their greater stability and an improved release [21,22,23,24]. In fact, there are plenty of works in the literature about adsorbed biomolecules in smectite clays [22,23,24,25,26,27,28,29] also studying the cargo–mineral interaction [30]. However, almost all of the mentioned works with delivery applications are based on a passive release mechanism [31], which usually consists of the delamination of the material by the solvent and the consequent release of the cargo. In contrast with this general passive mechanism, an active release can also be achieved, for instance, by functionalizing the external surface of the loaded support with a molecular gate or gatekeeper, which allows the on-command release of the cargo only when a selected stimulus is present. Although this mechanism has been widely used in mesoporous silica (MS) supports (known as gated MS), as far as the authors know, there are no similar examples using organonanoclays.

Therefore, it was our aim to demonstrate that it would be possible to prepare gated organonanoclays. As a proof of concept, we selected smectite clays as supports and oleic acid (OA) as a capping ensemble. Our choice of the gatekeeper was based on the fact that OA has demonstrated its ability to act as a molecular gate on mesoporous silica materials having different structures and pore sizes [32]. Moreover, it has been reported that OA-gated MS can deliver the entrapped cargo selectively in the presence of surfactants, such as bile salts, and are, therefore, excellent candidates to selectively deliver payloads in the small intestine (SI) where bile salts are present [4].

One group of molecules that could benefit from its protection in organonanoclay systems, are biomolecules that are too large to be encapsulated in other loadable materials, since clays are expandable and have no pore size restrictions [33]. As the entrapped molecules, we selected the dye rhodamine B (as the control system) and the bio-active molecules hematin (*Hem*) and cyanocobalamin (*B12*) which have estimated diameters of ca. 15–20 Å. Although previous works where *B12* is released from clays can be found in the literature [34,35], they are based on a passive release and do not have defined open-closed states that guarantee a zero background release in the absence of the stimulus. Furthermore, *Hem* and *B12* molecules are of special interest from a biochemical point of view. The *Hem* molecule contains an iron atom chelated into protoporphyrin IX, and *B12* is a vitamer of vitamin B_12_. These structural characteristics cause the these molecules to be related to iron deficiency anemia (IDA) [36]. In order to treat IDA, oral iron supplementation in the form of ferrous salts is normally the remedy used, but this could be associated with different side effects of varying severity [37]. Smart delivery systems for the controlled release of a more-bioavailable iron-form, like heme iron, could be a valuable option to effectively regulate iron levels without inducing overdose. Meanwhile, cobalamin (or vitamin B_12_) has a crucial role in cellular metabolism, and although its clinical deficiency is uncommon, subclinical deficiency affects up to 26% of the general population [38]. Its deficit is related to several problems [39,40,41,42] and it is usually due to malabsorption, especially in the elderly [43]. There are studies in the literature where high B12 doses orally administrated have been successful against pernicious anemia, a treatment also applicable to supplementation for intake-deficit problems [44,45]. However, the instability of the *B12* molecule under the adverse conditions of temperature, pH or light stipulates strict preservation processes, which seems to be a clear disadvantage [46,47]. Smart delivery systems for the controlled release of *B12* could be a good possibility to protect the *B12* molecule and release it in a controlled manner at its absorption place, the duodenum.

Based on the above, we report herein the use of Kunipia F (K_F_, natural refined montmorillonite), Sumecton SA (S_SA_, synthetic saponite) and Sumecton SWN (S_SWN_, synthetic hectorite) clays that were loaded with Rhodamine B (RhB), and S_SWN_ additionally loaded with Hem and B12, and capped with OA. These gated organonanoclays display “zero” payload releases in the absence of any stimulus, yet were able to deliver the cargo in the presence of surfactants, such as bile salts. In addition, the release experiments in the presence of bile salts were fitted to different mathematical models for drug release. The potential in vitro toxicity of the loaded and functionalized particles was also tested.

## 2. Materials and Methods

### 2.1. Chemicals and Cell Culture Media

Kunipia F (K_F_, natural refined montmorillonite), Sumecton SA (S_SA_, synthetic saponite) and Sumecton SWN (S_SWN_, synthetic hectorite) were acquired from Kunimine Industries Co. (Tokio, Japan). Rhodamine B (RhB), (3-aminopropyl)triethoxysilane (APTES), oleic acid (OA), N,N′-dicyclohexylcarbodiimide (DCC), hemin, cyanocobalamin (*B12*), cetyltrimethylammonium bromide (CTAB) and bile extract porcine were purchased from Sigma (Sigma-Aldrich Química S.L., Madrid, Spain). Ethanol (extra pure), hexane and dimethyl sulfoxide (DMSO) were acquired from Scharlab (Barcelona, Spain). In addition, 3-(4,5-dimethylthiazol-2-yl)-2,5-diphenyltetrazolium bromide (MTT) was provided by ThermoFisher (Madrid, Spain). Phosphate buffered saline (PBS), Dulbecco’s Modified Eagle’s Medium (DMEM), fetal bovine serum (FBS), penicillin/streptomycin antibiotic (P/S), non-essential aminoacids and all the needed mediums and materials for cell culture were provided by Labclinics (Labclinics S.A., Barcelona, Spain).

### 2.2. Gated Organonanoclays Synthesis

#### 2.2.1. RhB Loading

For model molecule (RhB) loading, 1 g of each clay (K_F_, S_SA_, S_SWN_) was added to 36 mL of a 10 mg/mL solution of RhB in water. Then the mixture was kept under stirring for 24 h. After that, the solids were centrifuged and dried at 40 °C. The obtained solids, loaded with RhB, were named K_F_-RhB, S_SA_-RhB and S_SWN_-RhB, respectively.

#### 2.2.2. Functionalization

With the aim of controlling payload release from the supports, the loaded clays were functionalized with organic moieties. Solid functionalization was performed in two steps; a preliminary adhesion of APTES as a linker molecule, and a final attachment of oleic acid to the APTES-amino-terminal groups through an amide bond. First step: APTES functionalization: For the first step of functionalization, 400 mg of each of the loaded clays (K_F_-RhB, S_SA_-RhB and S_SWN_-RhB) were resuspended in 15 mL of hexane and 2 mL of APTES were added, allowing the reaction to proceed for 5.5 h. After this process, the solids were isolated by centrifugation and dried under vacuum, obtaining the K_F_-RhB-N, S_SA_-RhB-N and S_SWN_-RhB-N materials. Second step: Oleic acid functionalization: Finally, to perform the last functionalization, 3 mL of OA was added to a solution of DCC in hexane (40 mg in 15 mL). After that, 300 mg of the appropriate APTES-functionalized solid (K_F_-RhB-N, S_SA_-RhB-N or S_SWN_-RhB-N) was added. The corresponding mixture was stirred for 20 h at room temperature and the synthesized solid was collected by centrifugation. The precipitate was washed with mixtures of ethanol:water with increasing proportions of water until no coloration was observed and finally dried under vacuum. The final gated organonanoclays, functionalized with OA, were K_F_-RhB-OA, S_SA_-RhB-OA and S_SWN_-RhB-OA, respectively.

### 2.3. Characterization Methods

X-ray diffraction (XRD), Field Emission Scanning Electron Microscopy (FESEM), N_2_ adsorption-desorption isotherms, zeta (ζ) potential measurements, infrared spectroscopy (FTIR), thermogravimetric analysis (TGA), Fluorescence spectroscopy and UV-Vis spectroscopy were employed to characterize the synthesized supports.

X-ray diffractograms were performed on a Bruker D8 Advance diffractometer (Bruker, Coventry, UK) using Cu Kα radiation. FESEM images were acquired with a Zeiss Ultra 55 (Carl Zeiss NTS GmbH, Oberkochen, Germany) and observed in the secondary electron mode. N_2_ adsorption-desorption isotherms were recorded with a Micromeritics TriStar II Plus automated analyzer (Micromeritics Instrument Corporation, Norcross, GA, USA). The samples were degassed at 120 °C in a vacuum overnight. The specific surface areas were calculated from the adsorption data in the low-pressure range using the BET model.

The functionalization process of all of the solids was followed by FTIR in a Bruker Tensor 27 FTIR spectrometer. To determine ζ potential values of the functionalized organonanoclays and their bare precursors, Zetasizer Nano ZS equipment (Malvern Instruments, Malvern, UK) was used. Samples were dispersed in ethanol at 20 °C, at a concentration of 1 mg/mL. Before each measurement, samples were sonicated for 2 min to preclude aggregation. ζ potential was calculated from the particle mobility values by applying the Smoluchowski model. The average of nine recordings was reported as ζ potential, and error bars were given with the standard deviation value.

The composition of loaded and functionalized clays was determined by TGA. Thermogravimetric analyses were carried out on a TGA/SDTA 851e Mettler Toledo balance (Mettler Toledo Inc., Schwarzenbach, Switzerland), using an oxidant atmosphere (air, 80 mL/min) with a heating program consisting of a heating ramp of 10 °C per minute from 20 to 1000 °C and two isothermal heating steps at 100 °C and 1000 °C for 30 min.

Fluorescence spectroscopy was carried out on a JASCO FP-8500 Spectrofluorometer (JASCO, Easton, Los Angeles, CA, United States) equipped with a FMP-825 Microplate reader and UV-Visible spectra were recorded with a JASCO V-650 spectrophotometer.

### 2.4. Cargo Delivery

To obtain the cargo release profiles of all of the model organonanoclays, 5 mg of the corresponding solids was placed in 10 mL of PBS (pH 7.5), simulating standard conditions in the gastrointestinal tract (GIT) and in 10 mL of PBS containing 10 mg/mL of bile extract, simulating specific surfactant conditions at the SI. At certain times (0.02, 0.25, 0.5, 1, 2, 4, 6, 8 and 24 h) aliquots of 700 µL were taken and filtered through 0.45 μm PTFE filters. Finally, the RhB content was determined by fluorescence spectroscopy. For the measurement, the employed excitation and emission wavelengths were 555 and 572 nm, respectively.

### 2.5. Cargo Release Kinetics

The payload release kinetics from all of the gated organonanoclays were calculated using different mathematical models. The selected models and their equations were Hill (1); Higuchi (2); Korsmeyer–Peppas (3):

% _Dye release_ = F_max_ · [t^γ^/(k^γ^ + t^γ^)], (1)

% _Dye release_ = k_H_ · t^½^*,* (2)

% _Dye release_ = k’ · t^n^ + b *a =* 1. (3)

### 2.6. Cell Culture Conditions

To test the possible toxicity of the different microdevices, Caco-2 human colon adenocarcinoma cells were selected as the model of GIT cells. Caco-2 cells were acquired from the American Type Culture Collection (ATCC) and cultured in DMEM medium supplemented with 10% FBS, 1% P/S and 1% non-essential aminoacids. The cells were maintained at 37 °C in a humidified atmosphere composed of 5% CO_2_ and 95% air. The cells underwent passage when they reached 80% confluence.

### 2.7. MTT Cell Viability Assay

Caco-2 cells were seeded at a density of 2 × 10^4^ cells/well in sterile 96-well plates and incubated for 24 h in the medium and the atmosphere conditions were as previously reported (see Section 2.6). All the solids under study were suspended in the concentrations to be evaluated (50, 100, 250 and 500 μg/mL). Each concentration was tested in 16 wells; being control cells without any solid. After 24 h of incubation, the cells were washed with PBS in order to remove the remaining particles. Then 100 μL of MTT solution in non-supplemented growing medium (0.5 mg/mL) was added to each well and the plates were incubated for another 2 h. After that, supernatant medium was removed and 100 μL of DMSO was added to each well. Finally, the plate was softly shacked until formazan crystals were completely solved, and the absorbance of the purple samples was measured at λ_exc_ = 550 nm.

## 3. Results and Discussion

### 3.1. Design, Synthesis and Characterization of Solids

The incorporation of gate-like ensembles into clays is an unexplored approach for controlled delivery applications. The development of responsive gated materials requires the selection of two components: (*i*) a suitable gate-like ensemble reactive to external stimuli and (*ii*) the selection of a structured matrix in which the gate-like assemble is grafted.

**Scheme 1:**
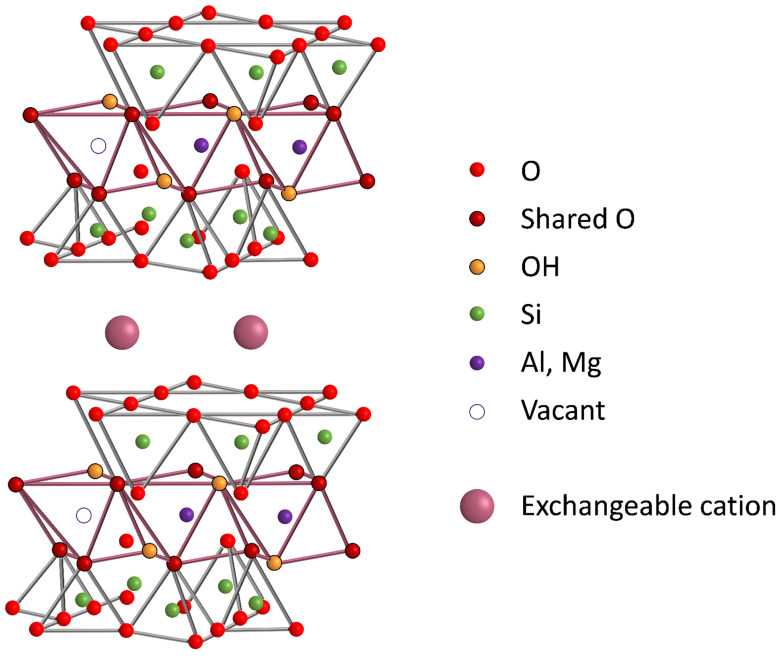
Representation of the laminar structure of smectite-clays.

Three different materials from the smectite clay-group (K_F_, S_SA_, S_SWN_) were selected as structured matrices taking advantage of their natural characteristics. The clay structure of the smectite clay-group is based on hydrated metallosilicate layers bonded by exchangeable cations (see Scheme 1). For the controlled delivery intended in this study, we have replaced these cations with different molecules (i.e., a dye and two bioactive molecules). Furthermore, the clay surface is similar to that of mesoporous silica and contains silanol groups that are easily modifiable by means of grafting methods, allowing the possibility of functionalizing clays with gate-like ensembles [48].

**Scheme 2:**
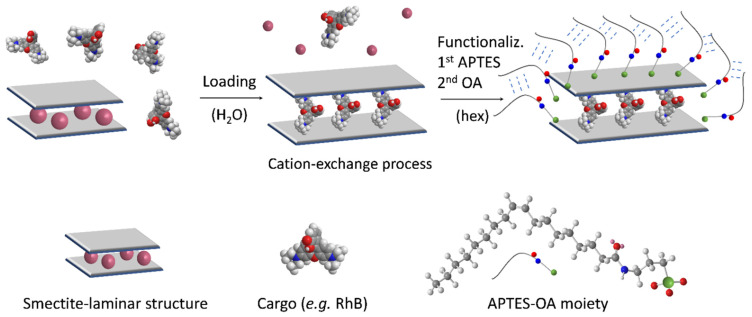
Representation of the synthesis of the gated clays. Cargo loading was produced by means of a cation-exchange process in an aqueous medium, whereby the guest molecule was intercalated between the layers of the smectite-clay. In the second step, the loaded support was functionalized with APTES and then with OA.

For the preparation of the gated materials, the clays K_F_, S_SA_ and S_SWN_ were first loaded with rhodamine B (RhB) following an immersion method by means of the exchange-cation capacity of these minerals. The reaction was maintained for 24 h to guarantee the clay delamination needed for cargo loading. In a second step, the loaded solids were reacted with APTES, and then oleic acid (OA) was attached to the APTES-amino-terminal group via the formation of an amide bond. The synthesis procedure is outlined in Scheme 2. We have recently reported that materials gated with fatty acids can be “opened” by interaction with surfactants. It has been suggested, for example, that such gated supports can be used for the controlled delivery of certain biomolecules in the small intestine (SI) due to the presence of bile salts (a natural surfactant). The OA gating mechanism has also been recently described and is due to the interaction forces between the hydrophobic tails of OA that act as a closing force, preventing cargo release from the system to an aqueous environment. However, in the presence of surfactants (such as bile salts) this interaction is interrupted, and the encapsulated payload is then allowed to be released. Once the loading and functionalization parameters were optimized with RhB, two more OA-gated S_SWN_ clays loaded with the bioactive molecules *Hem* and *B12* were prepared.

**Figure 1:**
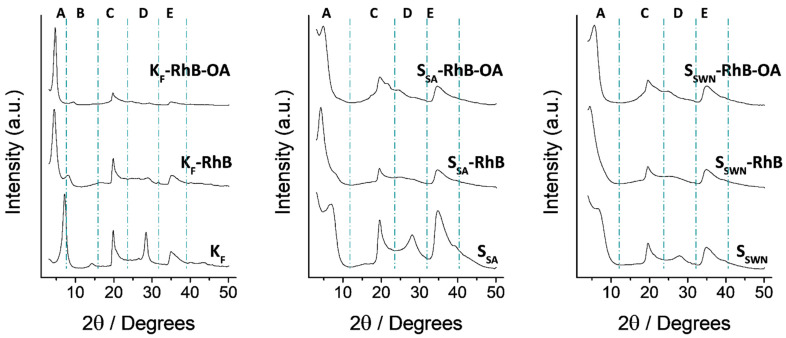
Normalized X-ray patterns of RhB-loaded organonanoclays in the different synthesis steps, indicated in their respective diffractograms. From bottom to top: bare, loaded and final gated material, respectively. The main XRD reflections are labelled from left to right as A–E, consecutively.

The synthesized particles were characterized by the usual standard techniques. Normalized X-ray diffraction patterns of bare, RhB-loaded and final gated clays are shown in Figure 1, and “d spacing” values of the more significant peaks in the solids are listed in Table 1. As it can be seen, these materials do not present very resolved patterns, and the diffraction diagrams are dominated by several intense peaks that correspond with the interlayer spacing. Hence, the first peak in each pattern would correspond with the distance between metallosilicate layers in the clays, and the other intense peaks are the corresponding higher order XRD reflections. Therefore, in all of the diffractograms, a low-angle region (3° to 10° 2θ) can be delimited where the intercalation of the different cargo molecules into the clay has its major effect. In this region, the most intense peak moves towards smaller 2θ values, i.e., higher d-spacing (see Table 1), when the guest molecule is effectively intercalated in the interlayer spacing, as this action produces the separation of the metallosilicate layers. However, OA functionalization of the different loaded solids does not produce a further increase but a shrinkage of the interlayer distance that could be related to some leak of the cargo and/or the interaction of OA moieties attached to the metallosilicate layers. Additionally, both the RhB intercalation and the OA functionalization produce a broadening of the other peaks as the intercalation process produces a loss of order in the structure.

**Figure 2:**
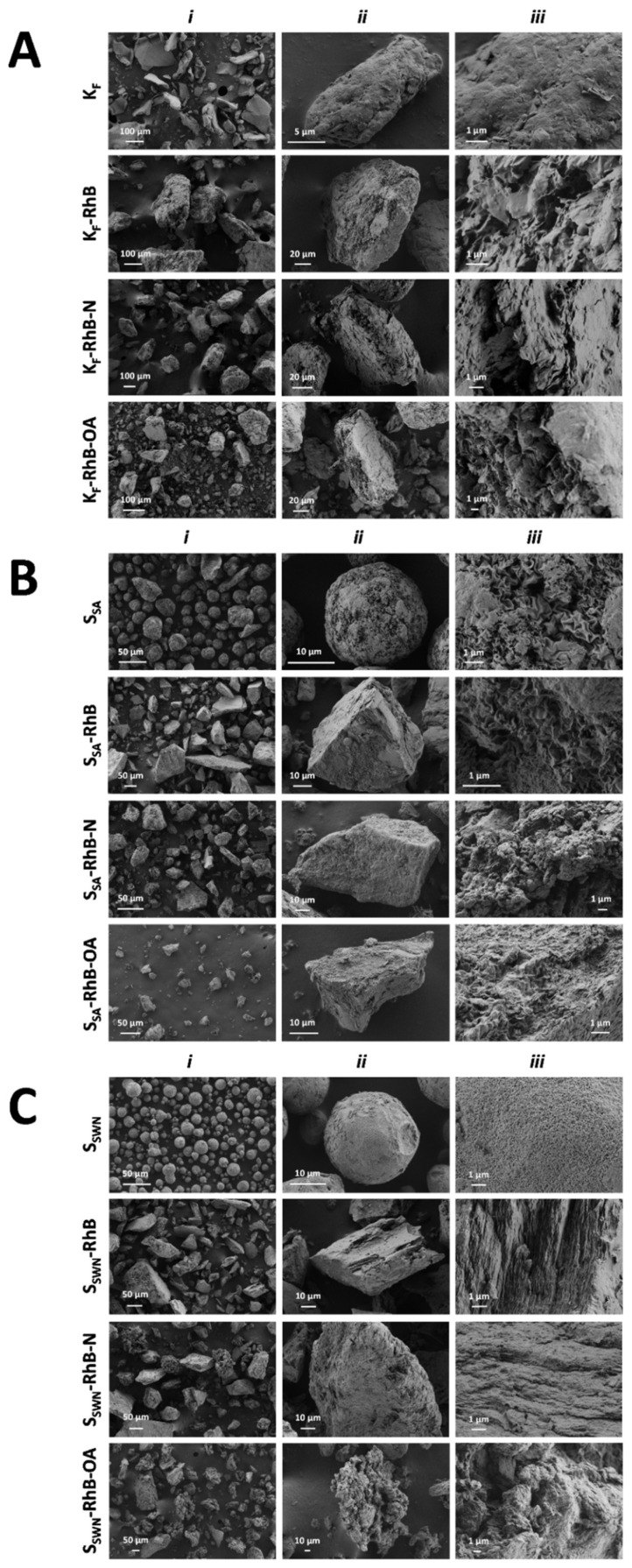
FESEM micrographs of the obtained solids in the different steps of synthesis of (**A**), K_F_-RhB-OA; (**B**), S_SA_-RhB-OA; and (**C**), S_SWN_-RhB-OA. General view of the particles (i), image of a representative particle (ii) and surface detail (iii).

FESEM images of all of the RhB-loaded final solids in the different synthesis steps are depicted in Figure 2 (K_F_ solids are shown in Figure 2A, S_SA_ solids in Figure 2B and S_SWN_ solids in Figure 2C). Starting from the bare materials (Figure 2, first row), as observed in the micrographs, the size of bare particles was quite similar for S_SA_ and S_SWN_ (Figure 2B(i),C(i)), that is on average up to 10–20 µm for S_SA_ particles and a slightly smaller size for S_SWN_ ones, whereas K_F_ had a tendency towards much larger agglomerates (Figure 2A(i)). However, the particle’s size was not the only difference among the chosen clay materials, as K_F_ particles showed quite an irregular shape, while S_SA_ showed more regular particles and S_SWN_ was mainly composed of spheres. This external shape of the different particles was related to a different microscopic arrangement of the metallosilicate plates in each one of the three solids. Thus, K_F_ particles showed a smooth surface (Figure 2A(iii)) that may be related to a microscopic parallel organization of its plaques, while both S_SA_ and S_SWN_ (Figure 2B(iii),C(iii), respectively) presented a microscopic pleated and folded arrangement of the plates to produce the corresponding rounded particle shape. These differences in the microscopic arrangement of the plates for the three solids could be related to the slightly higher crystalline order in the case of the K_F_ clay, which was shown in the smaller width of its XRD peaks.

After loading the rhodamine B molecule inside the three host clays (Figure 2, second row), changes in the microstructure were revealed (Figure 2A, K_F_-RhB; Figure 2B, S_SA_-RhB; and Figure 2C, S_SWN_-RhB). In regard to the particle shape, the biggest changes could be seen for the S_SA_ and S_SWN_ materials, as they completely lost their rounded shape and size homogeneity. In relation to the organization of the microscopic plaques, the three cases underwent important changes. While RhB-loaded K_F_ clay showed a rougher surface, bringing out a disordered arrangement of the microscopic plates, RhB-loaded S_SA_ clay still showed some domains of the pleated and folded microscopic arrangement and the RhB-loaded S_SWN_ clay showed quite a parallel organization of the microscopic plaques. These changes could be related to a delamination and reorganization process suffered during the immersion-based loading process.

The functionalization processes with APTES (Figure 2, third row) and finally OA (Figure 2, fourth row) did not significantly change the appearance of the particles, but increased the general disorder, making all of the materials at the end of the process quite similar in the less homogeneous distribution of particle sizes and the disordered arrangement of their internal microscopic plaques.

From the combination of FESEM and XRD data, it can be concluded that, despite the changes in size and shape of the obtained particles, the laminar structure of each material was not affected, since the XRD reflections of the initial material were maintained. Moreover, it can be determined that loading and functionalization processes modified the external appearance of the particles and increased the distance between clay layers due to the organic-matter inclusion into the interlamellar-space of the three materials.

**Figure 3:**
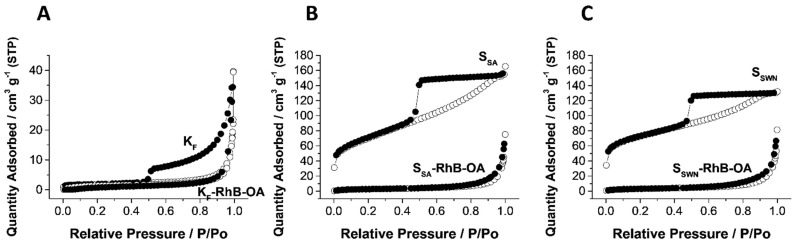
Nitrogen adsorption (○)—desorption (●) isotherms for all of the bare clays and final model gated organonanoclays. (**A**), K_F_ and K_F_-RhB-OA; (**B**), S_SA_ and S_SA_-RhB-OA; (**C**), S_SWN_ and S_SWN_-RhB-OA.

The N_2_ adsorption-desorption isotherms of the bare clays and the RhB-loaded final organonanoclays are shown in Figure 3, and the corresponding values of the specific surface and pore volume are collected in Table 2. Similar to in the case of the FESEM analysis, the three bare clays used as support in this work presented two differentiated behaviors. In one side, the K_F_ material presented a type II isotherm [49] that corresponded with a non-porous material. The very small knee at the low P/P_0_ values is related to the formation of the adsorbate monolayer and the thickness of the adsorbed multilayer (region of P/P_0_ close to one) has apparently no limit. Additionally, this support shows a hysteresis loop of type H3, that is typical of non-rigid aggregates of plate-like particles, what is clearly in agreement with the FESEM observations (vide supra). On the other hand, S_SA_ and S_SWN_ empty clays showed type IV isotherms with very similar features between them, which could be related to porosity in the mesopore range. Adsorption curves in both materials presented a more pronounced knee at the low P/P_0_ values than in the case of the K_F_ clay, corresponding with a higher specific surface (see Table 2). Moreover, in both cases the increment of the adsorbed N_2_ was produced very slowly along the full P/P_0_ range, ending only at values close to one in a very small plateau, corresponding with a very wide distribution of pore sizes (data not shown). S_SA_ and S_SWN_ clays also presented a hysteresis loop of type H2 that could be related to a percolation process of the liquid adsorbate through the clays’ structure. This different behavior between K_F_ clay on one side and S_SA_ and S_SWN_ on the other can be clearly related to their different microstucture revealed in the FESEM analysis. K_F_ presented smooth surface particles where the metallosilicate layers were quite well-ordered one on top of the other. However, S_SA_ and S_SWN_ presented particles where the metallosilicate plates are very corrugated, producing the spherical microparticles observed in the FESEM micrographs (Figure 2A–C, first row). The corrugation of the structural layers must have been the responsible of this mesoporosity with a very wide pore size distribution. During the loading and functionalization processes, the clays suffer consecutive delamination and reorganization of the metallosilicate layers. Therefore, all the three final materials presented similar non-corrugated layers in the FESEM micrographs (see Figure 2) and hence, the three of them only showed gas adsorption in the region of high P/P_0_ values, which corresponded to intraparticle pores. Consequently, while an almost-negligible change in specific surface area and pore volume was detected for the K_F_ clay, the S_SA_ and S_SWN_ ones suffered an important decrease in the organonanoclay N_2_-adsorbed volume and surface area reaching the values of the K_F_ support (see Table 2). Hence, the increase in the value of the d-spacing of the first peak in the three loaded materials (Table 1, peak A) is directly related to the loading of the RhB molecule inside the interlayer spacing.

**Figure 4:**
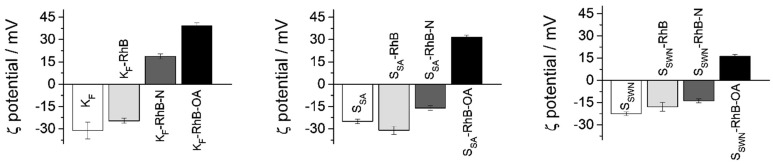
Zeta (ζ) potential values of all of the model organonanoclays. The increasing darkness in bar colors corresponds to the progressive synthesis steps: from bare clays to loaded, APTES-functionalized and final OA-functionalized organonanoclays (white, light gray, dark gray and black bars, respectively).

Loading and functionalization processes which led to the final gated solids were followed by zeta (ζ) potential. The measurement assay was performed in EtOH at 20 °C and the obtained values are reported in Figure 4. As it can be observed, the three bare clays (white bars) exhibited negative ζ potential values (between −20 mV and −30 mV). After the loading process, the charge of the materials remained negative (light gray bars) since the external groups of the material were not modified. Then, once the APTES molecule was added, ζ potential values became positive (K_F_) or less negative (S_SA_ and S_SWN_) (dark gray bars), confirming the efficiency of the functionalization process. The neutralization of the charge is a direct consequence of the presence of the amino group of APTES on the surface. Finally, the anchoring of OA to the APTES moieties increased the positive charge of the particles, resulting in positive ζ potential values for all of the model organonanoclays.

**Figure 5:**
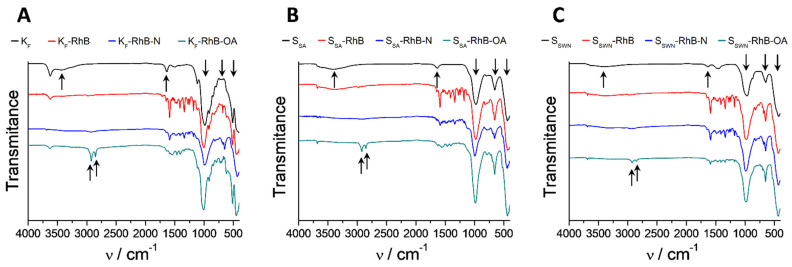
FTIR spectra of the consecutive synthesis steps of the different model organonanoclays: (**A**), K_F_-RhB-OA; (**B**), S_SA_-RhB-OA; (**C**), S_SWN_-RhB-OA.

All the RhB-loaded organonanoclays in the different synthesis steps were also characterized by infrared spectroscopy (FTIR) and the obtained spectra are depicted in Figure 5. The dominant bands in all of the FTIR spectra are those which belong to the silica tetrahedra and metal octahedra, which conform to the laminar structure of all of the phyllosilicates (1050, 600 and 450 cm^−1^). All the bare clay precursors are part of the smectite family, and as result of the structural resemblance, the FTIR spectra thereof are similar for the three of them. The broad band that appeared around 3500 cm^−1^ was related to the vibration of the hydration water molecules and it disappeared during the functionalization process. The small but sharp band at 3600 cm^−1^ and the sharp one at 1600 cm^−1^ were assigned to the hydroxyl group coordinated to octahedral cations, mainly Al^3+^ in the K_F_ clay (montmorillonite) and Mg^2+^ in S_SA_ (saponite) and S_SWN_ (hectorite) clays. The clear appearance of two bands at approximately 2900 cm^−1^ and 2850 cm^−1^ in all of the spectra of the gated clays was assignable to the bending C–H vibrations. These new bands were related to the presence of APTES and OA [4].

The contents (α_i_) in mmol·g^−1^ of the remaining residue (metal oxides from the inorganic support) of cargo, APTES and OA in the final RhB loaded organonanoclays were determined by TGA and are shown in Table 3. As it can be seen, the three materials presented similar amounts of loaded RhB but different degrees of gate functionalization. The relationship between the organic matter content, functionalization percentage and payload release is explained in Section 3.2 (vide infra).

### 3.2. Cargo Controlled Release

In order to evaluate the feasibility of different supports to control the bioaccessibility of the payload, delivery studies of RhB from the three loaded and functionalized clays were carried out in the absence and presence of bile salts. RhB concentrations in the different aliquots were measured by means of fluorescence spectroscopy.

**Figure 6:**
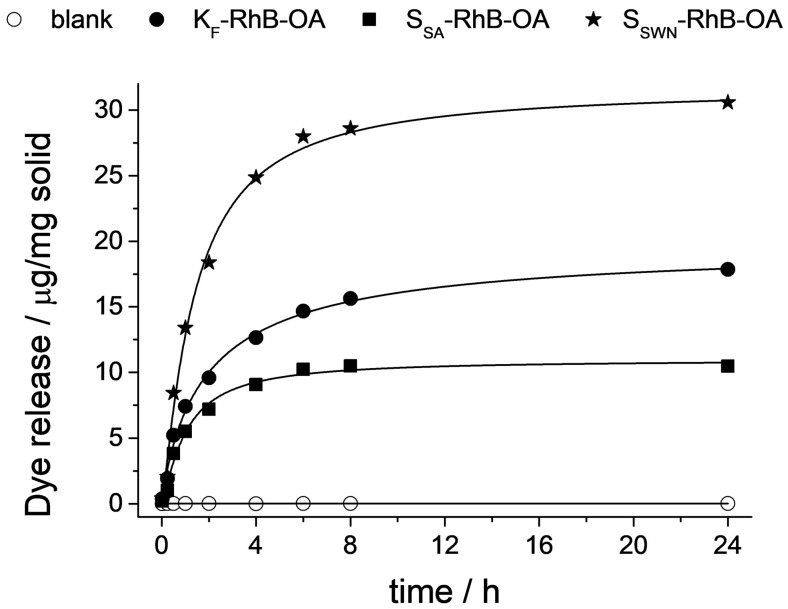
Release profile of RhB dye from all of the gated organonanoclays in PBS (○) and in the presence of bile salts (● K_F_-RhB-OA, ■ S_SA_-RhB-OA, ★ S_SWN_-RhB-OA).

Figure 6 shows the release behavior of the three gated clays loaded with the cited fluorophore and functionalized with OA. For all of the three systems, a zero baseline was found in release experiments in PBS in the absence of bile salts, indicating that RhB remained inside the layers of the clays and it was not released to the solution. In contrast, in the presence of bile salts (conditions of the SI), a progressive delivery of RhB was observed for all of them. This different and remarkable behavior in the presence of the triggering stimulus, when compared with that of the PBS alone, was due to the surfactant effect of bile salts. This fact confirmed the ability of OA to act as a molecular gate when anchored on these clays, even when they had a layered structure with an expandable interlayer distance, compared with other mesoporous materials typically used for the design of controlled delivery systems. Comparing release curves of the different organonanoclays, it can be stated that S_SWN_ is the material with the highest delivery capacity, followed by K_F_ and S_SA_. To compare the different releasing capacities of the synthesized materials, the maximum payload released must be related to the organic matter content of each solid (Table 3, vide supra). Considering the data included in Table 3, the different functionalization of the three composites, combined with the structural differences of the host matrices, could be responsible for the different release capacities presented by the organonanoclays. Thus, while hectorite (S_SWN_) and montmorillonite (K_F_) clays presented substitution in the octahedral (Al/Mg) positions of the metallosilicate layers, saponite clay (S_SA_) presented substitution in the tetrahedral (Si) positions. This substitution at the tetrahedral sites may produce higher interaction between the metallosilicate layers and the cationic RhB moieties. Then, the material with the highest release (S_SWN_) is the one with the lowest gate functionalization and octahedral substitution, whereas the one with the lowest release (S_SA_) corresponds with an intermediate gate functionalization and tetrahedral substitution. Montmorillonite (K_F_) clay presented, as with hectorite (S_SWN_) clay, octahedral sites substitution, but a higher functionalization was what halved the final release for this material compared to the one of the hectorite.

Moreover, in order to investigate the release of RhB dye from all of the gated organonanoclays in the stomach, a release experiment in gastric pH condition was performed. As shown in Figure S4, all of the gated organonanoclays exhibited non-cargo release when exposed at pH 2.5 and confirmed the suitability of the OA molecular gate to hinder the release in the absence of the proposed stimulus.

Once the loading and functionalization parameters were optimized with the model molecule (RhB), the synthesis and release kinetics experiments of clays loaded with bioactive molecules were carried out. Two biomolecules, *Hem* and *B12*, were selected to be encapsulated in the clay with the highest delivery capacity: i.e., S_SWN_. The synthesis of the bioactive organonanoclays was followed using XRD, ζ potential, FTIR and TGA techniques (data in Supporting Information: Figures S1–S3). In general, bioactive-loaded clays showed similar results to those found in the RhB-loaded materials. However, it is worth pointing out some differences in the evolution of the XRD diagrams when loading the S_SWN_ clay with both biomolecules. In first place, the intercalation of *Hem* and *B12* in the chosen clay produced a lower displacement of the first peak than the one observed for the S_SWN_-RhB material, but was similar for both biomolecules (from 13.55 Å to 14.52 Å and 14.65 Å for *Hem* and *B12* molecules, respectively). However, the functionalization with OA showed quite a different result for both materials. In the case of the B12-loaded clay, the final functionalization with OA produced a smaller, almost indiscernible decrease of the interlayer distance when compared with the RhB-loaded materials, so no leak of the loaded biomolecule during the final functionalization steps had taken place. However, in the case of the Hem-loaded S_SWN_ material, not only was there no decrease in the interlayer distance but there was also a further increment in the value of the interlayer separation. This different behavior could be related to a possible intercalation of the OA molecules in the interlayer spacing, since hematin molecules were already present in the interlayer spacing of the clay.

**Figure 7:**
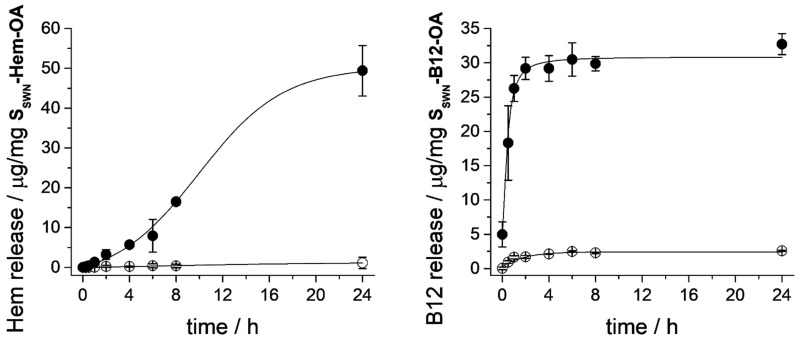
Release profiles of *Hem* (**left**) and *B12* (**right**) from S_SWN_-Hem-OA and S_SWN_-B12-OA organonanoclays, respectively. Suspensions of the gated supports in PBS (○), and in a solution containing CTAB-surfactant molecules (●).

Once the S_SWN_-Hem-OA and S_SWN_-B12-OA materials were characterized, the procedure followed to obtain the release profiles of the loaded biomolecules was similar to that used in the case of the RhB-loaded organonanoclays. The details are included in the Supporting Information and the obtained profiles are shown in Figure 7. Both supports had zero or negligible release when they were suspended in PBS media and a marked payload release was produced when surfactant molecules were present (for more information about release kinetics, see Section 3.3). This demonstrated that OA-moiety also efficiently closed the phyllosilicate-based system when large molecules were the entrapped cargo between metallosilicate layers, and that the OA gate allowed the release of these large molecules when the appropriate stimulus (e.g., bile salts) was present. However, some differences could be observed between the release profile of these two materials. In the case of the B12-loaded clay, the cargo was quickly released, reaching the maximum payload after 4 h. In the case of the Hem-loaded solid, the release of the cargo was considerably more sustained in time, and it only reached its maximum payload after 24 h. Taking into account the differences in the XRD diagrams of both functionalized materials (see above) we may relate this different behavior to the presence of OA also intercalated in the interlayer spacing that would produce a more complex release process.

Despite the fact that *B12* has previously been encapsulated in similar supports [34,35], this is the first time that *B12* and *Hem* were protected in a system with a molecular gate that effectively released them in an active and controlled way. The zero-release achieved by all of the synthesized organonanoclays, regardless of the cargo-molecule-size, constituted an important outcome in relation to the functionality of OA as a molecular gate.

Moreover, in order to highlight the effect of OA, release experiments of smectite clays without OA coating have been developed, showing the burst release when non-OA were covering the organonanoclays (Figures S5–S7).

### 3.3. Cargo Release Kinetics

Besides achieving a remarkable surfactant-triggered release of the cargo, it was also our aim to evaluate the capability of those systems to control the delivery of the payload molecule over time. With this purpose, data from release experiments in the presence of bile salts were fitted to different mathematical models for drug release: Hill [50], Higuchi and Korsmeyer–Peppas [51].

The Hill equation has been widely employed to fit data from different physicochemical phenomena as enzymatic reactions or the relation between drug concentration and its effect. The main reason for its success is probably its flexibility and effectiveness in fitting experimental data. In our case, the Hill equation would allow us to simulate sigmoidal release patterns in which there is an initial slow release, followed by a faster intermediate release, and finally a further slow release. The fitting of the experimental values to this model has allowed us to calculate parameters as the maximum payload release (F_max_), the sigmoicity coefficient of the curve (γ) and the constant k related to the time needed to get 50% of release, which can help us to compare the different releasing processes.

The Higuchi model is based on Fickian diffusion processes taking into account three hypotheses: (i) the initial drug concentration in the matrix is much higher than the drug solubility, (ii) the drug diffusion takes place only in one dimension and, (iii) the drug diffusivity is constant. From the fit of the data to this model, Higuchi’s constant K_H_, related to the velocity of diffusion of the drug from the supporting matrix, can be obtained.

The semi-empirical model described by Korsmeyer–Peppas (K–P), also denominated “power law”, adds some modifications to the Higuchi model and makes it less restrictive. It is applied to drug release from matrices in which several phenomena occur simultaneously, not only diffusion. Three different parameters are obtained from the mathematical adjustment of the data to the power law that can give us important information about the cargo release process. In the first place, *K* is a constant that is directly related to the interaction between the cargo and the host matrix, i.e., the larger the value of the constant, the bigger the interaction. Then, the parameter *b* indicates the presence or not of a phenomenon denominated “burst release” that indicates the immediate release of the load in the corresponding medium. Finally, the parameter *n*, that is the more complex one, is related to the type of diffusion. For a cylindrical geometry of the support, the value for this constant should be *n = 0.45* in the case of Fickian diffusion, but it would have larger values if the system presented non-Fickian diffusion. In this last case, there are three possibilities which differ in the velocity of the solvent diffusion (*V_s-d_*) with respect to the polymeric relaxation process (*P_r_*). The possibilities are (*i*) 0.45 *< n* *<* 0.89 if there is anomalous transport, where *V_s-d_* and *P_r_* have similar magnitudes; (*ii*) n = 0.89 defines the Case I transport, where *V_s-d_* < *P_r_*; and (*iii*) *n* > 0.89 indicates Super Case II transport, where *V_s-d_* >> *P_r_* which causes an acceleration of solvent penetration [51]. Although the release process from the organonanoclays designed in this study is not produced from a polymeric matrix, it is also possible to obtain information about the release mechanism in our materials as the solvent will have to diffuse inside the clay composites to extract the cargo molecules (*V_s-d_*), against the gating moieties that should suffer the corresponding relaxation process (*P_r_*) when the external stimulus is present. The values of all of the parameters calculated from the release kinetics data of all of the synthesized organonanoclays with the aforementioned mathematical models, as well as the obtained coefficients of determination (r^2^), are listed in Table 4.

The Hill model is the most flexible of those proposed, and hence it offers the best adjustment of the release kinetics of the five materials, allowing us to get a first comparison among the solids. From the values in Table 4 we can see that the three materials loaded with RhB presented quite similar parameters. The material with a higher F_max_ value was S_SWN_-RhB-OA, as it corresponded with the highest cargo loading, followed by K_F_-RhB-OA and S_SA_-RhB-OA, which presented similar F_max_ values. In the case of the *B12* release from S_SWN_-B12-OA, the obtained parameters were similar to those of the RhB-loaded solids, but it presented smaller values for k and γ, which indicate a faster release of the *B12* vitamin. The *Hem*-release curve is the most different of all, showing larger values for both k and γ parameters, which indicate a slower and maintained release of the cargo.

The adjustment of the data with the Higuchi model provides worse results, being the coefficients of determination especially low for the S_SWN_-Hem-OA and S_SWN_-B12-OA materials (r^2^ < 0.9). Although the fit of the release kinetics data of these two materials with the Higuchi model is not perfect, the value of the K_H_ constants obtained indicated that the *B12* release was the fastest process of those studied, while the *Hem* release was the slowest one. These premises are in agreement with the information obtained from the Hill model.

Finally, the Korsmeyer–Peppas model shows a very good adjustment of the kinetics for the three RhB loaded materials. The free refinement of the “n” parameter gives values quite close to 0.45, indicating a Fickian diffusion for the RhB release. The three materials also presented a zero value for the “b” parameter, indicating a null burst release, and similar values for the “K” parameter, which would indicate a similar interaction between the RhB cation and each of the three host matrices. Restricting the “n” value for these materials to the range imposed by the K–P model’s definition (n ≥ 0.45), the adjustment was still quite good, and only small changes in the parameters (b and K) were obtained, whereas the relative order of the values was maintained (see supplementary material, Table S2).

Although the application of the K–P model to the release of the *B12* molecule offers poor adjustment, the obtained n and K parameters were similar to the ones of the RhB composites. This fact would also indicate a Fickian diffusion of the cargo, however in this case a big burst release was produced. On the other hand, when the K–P model was applied to the release of the *Hem* molecule we obtained a better adjustment with quite a different parameter collection. In first place, the *n* parameter obtained the value of 1.21, which indicated a Super-Case II non-Fickian release (*V_s-d_* >> *P_r_*), with a fast solvent penetration and governed by the swelling of the composite. This result would be in agreement with a more complex OA functionalization in the S_SWN_-Hem-OA material, revealed by its large increment of the interlamellar spacing after the last synthesis step compared with the other materials (see XRD data in Table 1 and Table S1). Additionally, the value of the K parameter was very low, indicating a very small interaction of the *Hem* moiety with the S_SWN_ matrix, markedly lower than the one found in the case of the RhB or *B12* molecules. This is because the anionic charge of the aluminosilicate layers of the three clay materials would interact with higher strength with RhB and *B12* compared with *Hem*, since the first two molecules have cationic character while the *Hem* molecule should present an anionic charge at the pH of the loading process.

### 3.4. In Vitro Biocompatibility Test: Interaction with Cells of the GIT

In addition to the cargo release experiments from the five different organonanoclays, it was another aim of this study to assess the biocompatibility of the developed systems. Therefore, cell viability studies with all of the bare phyllosilicates and final loaded-and-functionalized solids were performed. Caco-2 human colon carcinoma cells were selected to evaluate a possible toxic effect of the cited microdevices on cells of the GIT. Similar studies found in the literature report low toxicity for this kind of materials at standard concentrations (ca. 50 μg/mL) [19], which was the reason that chronic particle concentrations were tested in the current assay (up to ca. 500 μg/mL). The cells were treated with each one of the prepared systems for 24 h at concentrations of 50, 100, 250 and 500 μg/mL. After this time, a cell viability assay using MTT was performed, which was based on the absorbance measurement of formazan produced by oxidoreductase enzymes of viable cells [52]. The formazan crystals produced by mitochondrial respiration were insoluble in the growing medium, so DMSO solvent needed to be added after medium removal in order to solubilize the generated compound. The absorbance of the resulting purple solution was measured at λ_exc_ = 550 nm and compared with the one achieved by the control cells.

**Figure 8:**
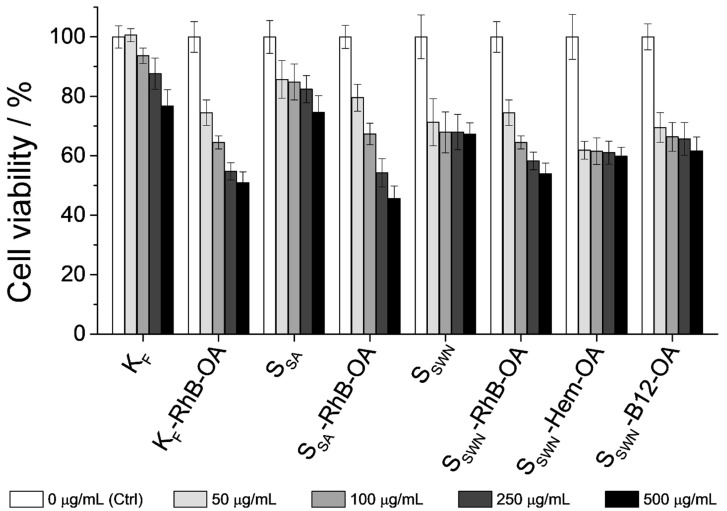
MTT cell viability assay of Caco-2 cells exposed to the indicated materials. Caco-2 cells were treated with the bare clays and the gated organonanoclays at concentrations of 0 (control), 50, 100, 250 and 500 μg/mL (increasing from light grey to black) for 24 h. Cell viability was quantified by means of DMSO solubilization of the MTT-metabolization crystals.

Figure 8 shows the obtained results from the MTT cell viability assay. First of all, focusing on bare materials toxicity, K_F_ was the least toxic one, followed by S_SA_ and finally S_SWN_. If the average particle size observed in FESEM micrographs was compared, K_F_ aggregates were much larger than S_SA_, and these were slightly larger than S_SWN_ (see Section 3.1 for details). Therefore, it can be deduced that the larger the particle size, the lower the material’s toxicity. These results agree with multiple data in the literature, because large particle sizes partially prevent their cellular internalization [53]. An increase in the toxicity levels was observed at high particle concentrations, probably because the cell monolayer became overspread due to the sedimentation of the particles. This fact has been previously described in other works in the literature [54], and the increased toxicity at high concentrations observed in the present work may be related to this circumstance.

The toxicity of all of the loaded and functionalized organonanoclays was comparable between them, which may be related to their similar average size and functionalization. The cell viability values were very similar to each other and were slightly lower than those of the bare precursors. Focusing on particle concentrations, standard amounts of the tested organonanoclays (ca. 50 μg/mL) retained cell viability at high levels (higher than 70%). The results also showed that even at particle concentrations as high as 500 μg/mL, S_SWN_ solids (S_SWN_-RhB-OA, S_SWN_-Hem-OA and S_SWN_-B12-OA) were as biocompatible as the starting bare material.

## 4. Conclusions

Three different phyllosilicates from the smectite clay-group (montmorillonite, saponite and hectorite) were successfully loaded with different cargos (rhodamine B, hematin and cyanocobalamin) and functionalized with oleic acid as a molecular gate. The new hybrid organonanoclays were capable of entrapping these molecules and efficiently delivering them in an aqueous environment only when surfactant agents (e.g., bile salts) were present. The cargo release profile of all of the synthesized organonanoclays was studied by fitting the data with three different release kinetic models: Hill, Higuchi and Korsmeyer–Peppas (K–P) models. The most instructive result was obtained with the K–P model, which highlighted the dependence of release kinetics not only on the organic–inorganic hybrid system but also on the nature of loaded molecules and their interaction with the support. With the aim of testing all of the synthesized organonanoclays as cargo release microdevices, in vitro cell viability assays were performed with Caco-2 adenocarcinoma cells. The obtained results from this assay showed that particle concentrations of ca. 50 μg/mL maintained cell viability at good levels (higher than 70%), demonstrating that the studied organonanoclays were well tolerated by cells at these concentrations. This work demonstrated the effectiveness of oleic acid as a molecular gate for three different clay materials, allowing active and controlled release of the entrapped cargo. Moreover, the zero-release achieved by this molecular gate opens its use to deliver drugs that must be only released in the intestinal endothelium, where bile salts are present, as target tissue and further works will be developed in due course.

## Figures and Tables

**Table 1:** Main reflections’ d-Spacing (Å) values for the RhB-loaded organonanoclays obtained from Bragg’s law (nλ = 2d · sin(θ), λ = CuK_α av_ = 1.54 Å, *n* = 1).

	A	B	C	D	E
K_F_	12.35	6.22	4.48	3.13	2.56
K_F_-RhB	19.61	11.47	4.48	3.14	2.54
K_F_-RhB-OA	18.79	9.40	4.49	3.13	2.56
S_SA_	12.35		4.55	3.19	2.58
S_SA_-RhB	21.71		4.55		2.59
S_SA_-RhB-OA	18.79		4.57		2.58
S_SWN_	13.55		4.52	3.21	2.57
S_SWN_-RhB	19.96		4.54		2.57
S_SWN_-RhB-OA	15.64		4.55		2.56

**Table 2:** BET specific surface values and pore volumes calculated from the N_2_ adsorption-desorption isotherms for selected materials.

	S_BET_ (m^2^ g^−1^)	Pore Volume ^a^ (cm^3^ g^−1^)
K_F_	5.7	0.04
K_F_-RhB-OA	4.8	0.03
S_SA_	250.5	0.14
S_SA_-RhB-OA	9.9	0.01
S_SWN_	242.1	0.10
S_SWN_-RhB-OA	11.3	0.07

^a^ Pore volumes are restricted to the intraparticle mesopore area.

**Table 3:** Content (α) in mmol g^−1^ residue (after calcination) of cargo, APTES and OA in the final organonanoclays, determined by TGA.

Solid	α_cargo_	α_APTES_	α_OA_
K_F_-RhB-OA	0.69	1.3	1.3
S_SA_-RhB-OA	0.74	0.79	0.61
S_SWN_-RhB-OA	0.78	0.31	0.30

**Table 4:** Parameters and coefficients of determination (r^2^) obtained by adjusting the data to each model Equations (1)–(3).

System	Hill				Higuchi		K–P			
	F_max_	γ	k (min)	r^2^	K_H_ (min^−½^)	r^2^	K (min^−n^)	n	b	r^2^
K_F_-RhB-OA	17.85	0.96	86.14	0.991	0.78	0.97	7.75	0.40	0	0.99
S_SA_-RhB-OA	10.47	1.27	52.54	0.987	0.55	0.94	11.1	0.37	0	0.98
S_SWN_-RhB-OA	30.57	1.21	73.64	0.995	1.46	0.96	7.83	0.41	0	0.98
S_SWN_-Hem-OA	49.38	1.83	750.02	0.948	0.50	0.83	0.01	1.21	0.71	0.96
S_SWN_-B12-OA	32.72	0.79	14.86	0.975	1.79	0.69	3.49	0.42	44.2	0.74

## Data Availability

Not applicable.

## Supplementary Materials

The following supporting information can be downloaded at: https://www.mdpi.com/article/10.3390/nano12152694/s1, Figure S1: Normalized X-ray patterns of the bioactive organonanoclays (indicated in their respective diffractogram). From bottom to top: bare, loaded and final gated organonanoclay, respectively. The main XRD reflections are labelled from left to right as A–E, consecutively. B-peak is omitted in order to correlate the peaks with the previously obtained for model-solids (Figure 1); Figure S2: Zeta (ζ) potential values of all the bioactive organonanoclays. The increasing darkness in bar colors corresponds to the progressive synthesis steps: from bare S_SWN_ to loaded, APTES-functionalized and final OA-functionalized organonanoclays (white, light gray, dark gray and black bars, respectively); Figure S3: FTIR spectra of the consecutive synthesis steps of the bioactive organonanoclays: A, S_SWN_-Hem-OA; B, S_SWN_-B12-OA; Figure S4: Release profile of RhB dye at pH 2.5; Figure S5: Release profile of RhB dye of smectite clays without OA coating at pH 7.5; Figure S6: Release profiles of Hem at pH 7.5 from SSWN-Hem; Figure S7: Release profiles of B12 at pH 7.5 from, SSWN-B12; Table S1: Main reflections’ d-Spacing (Å) values for the bioactive organonanoclays obtained from Bragg’s law (nλ = 2d · sin(θ), λ = CuK_α av_ = 1.54 Å, *n* = 1); Table S2: Parameters and coefficients of determination (r^2^) obtained by adjusting the data with the K–P model (Equation (3)) fixing the value of the *n* parameter (*n* ≥ 0.45).
