# Supplementary material for: Gated Organonanoclays for Large Biomolecules: Controlled Release Triggered by Surfactant Stimulus
Source: Nanomaterials (Basel). 2022 Aug 5;12(15):2694. doi: 10.3390/nano12152694 (PMC9370449; doi:10.3390/nano12152694)
Supplement: Supplementary file 1 [file nanomaterials-12-02694-s001.zip › nanomaterials-1830903-supplementary.pdf]

SUPPORTING INFORMATION

GATED-ORGANONANOCLAYS FOR LARGE BIOMOLECULES CONTROLLED RELEASE TRIGGERED BY SURFACTANT STIMULUS

Elisa Poyatos-Racionero,[a,b] Édgar Pérez-Esteve,[c] Serena Medaglia,[a,b] Elena Aznar,[b,a,d,e] José M. Barat,[c] Ramón Martínez-Máñez,[a,b,d,e,f] M. Dolores Marcos[a,b,d,e,f]* and Andrea Bernardos[b,a,e]*

[a] Instituto Interuniversitario de Investigación de Reconocimiento Molecular y Desarrollo Tecnológico (IDM). Universitat Politècnica de València, Universitat de València. Camino de Vera s/n, 46022, Valencia (Spain)

[b] CIBER de Bioingeniería, Biomateriales y Nanomedicina (CIBER-BBN) (Spain)

[c] Departamento de Tecnología de Alimentos, Universitat Politècnica de València. Camino de Vera s/n, 46022, Valencia (Spain)

[d] Unidad Mixta de Investigación en Nanomedicina y Sensores. Universitat Politècnica de València, IIS La Fe de Valencia 46026, Valencia (Spain)

[e] Unidad Mixta UPV CIPF Invest Mecanismos Enfermedad. Universitat Politècnica de València, Ctr Invest Príncipe Felipe 46100, Valencia (Spain)

[f] Departamento de Química, Universitat Politècnica de València. Camino de Vera s/n, 46022, Valencia (Spain)

Bioactive organonanoclays synthesis

In order to derivatize hemin into hematin, the Cl^- ligand coordinated to the central Fe atom was replaced by an OH^- ligand in aqueous solution. With this aim, 6 mL of 0.5 N NaOH were added to 20 mL of H_2O, and then 650 mg of hemin were dissolved in the basified H_2O. To carry out the loading of S_{SWN} with Hem, 1 g of S_{SWN} was added to the aforementioned solution, and the mixture was stirred overnight. The final solid was collected by centrifugation and dried at 40 °C (S_{SWN}-Hem). To perform the APTES-functionalization, 400 mg of S_{SWN}-Hem were suspended in 15 mL of hexane and 2 mL of APTES were added. The mixture was stirred at room temperature for 5.5 h, and the final solid (S_{SWN}-Hem-N) was obtained by centrifugation and dried under vacuum. To perform the OA-functionalization, 300 mg of S_{SWN}-Hem-N were added to a previous solution composed by 3 mL of OA in hexane (15 mL) and 40 mg of DCC. The mixture was stirred overnight, and the precipitate was collected by centrifugation and washed with EtOH:H_2O mixtures with increasing amounts of H_2O until colorless-supernatant was obtained. The final solid (S_{SWN}-Hem-OA) was centrifuged and dried in vacuum.

The procedure followed to obtain the second bioactive microdevice, loaded with *B12*, was similar to the one used for Hem. For the cargo loading, 1 g of S_{SWN} was added to a solution of *B12* in water (100 mg in 10 mL) to obtain S_{SWN}-B12 solid. The APTES-functionalization and posterior OA-functionalization procedures, as well as the reagents proportions, were the same as the employed with the Hem-loaded-solids (*vide supra*). Accordingly, S_{SWN}-B12-N solid (APTES functionalized) and S_{SWN}-B12-OA solid (OA functionalized) were obtained.

Bioactive organonanoclays cargo delivery

The procedure followed to obtain cargo delivery profiles of the bioactive molecules was similar to the one used for the model solids (see section 2.4). However, as it was done in previous works [4], bile salts were replaced by another surfactant that had no background signal in the range where the bioactive molecules have their maximum absorption. For this reason, 5 mg of the corresponding solid were placed in 10 mL of PBS as blank (simulating conditions in absence of bile salts) and a solution of surfactant agent in PBS ($5 \cdot 10^{-3}$ M CTAB in PBS) as stimulus. At certain times (0.02, 0.25, 0.5, 1, 2, 4, 6, 8, 24 h) aliquots of 700 µL were centrifuged and the bioactive molecule content determined by means of its absorbance in a UV-Vis spectrophotometer. For the measurement, the employed excitation wavelengths for *Hem* and *B12* were 385 and 361 nm, respectively.

Bioactive organonanoclays characterization

The synthesized bioactive organonanoclays were characterized with the usual techniques. X-ray diffraction (XRD), infrared spectroscopy (FTIR), zeta (ζ) potential measurements and thermogravimetric analysis (TGA) were performed.

Normalized X-ray patterns of bare, loaded and final gated-clay are shown in Figure S1. These materials present low resolution patterns dominated by the intense peaks related to the metallosilcate interlayer spacing. The “d spacing” values of the more significant peaks are listed in Table S1.

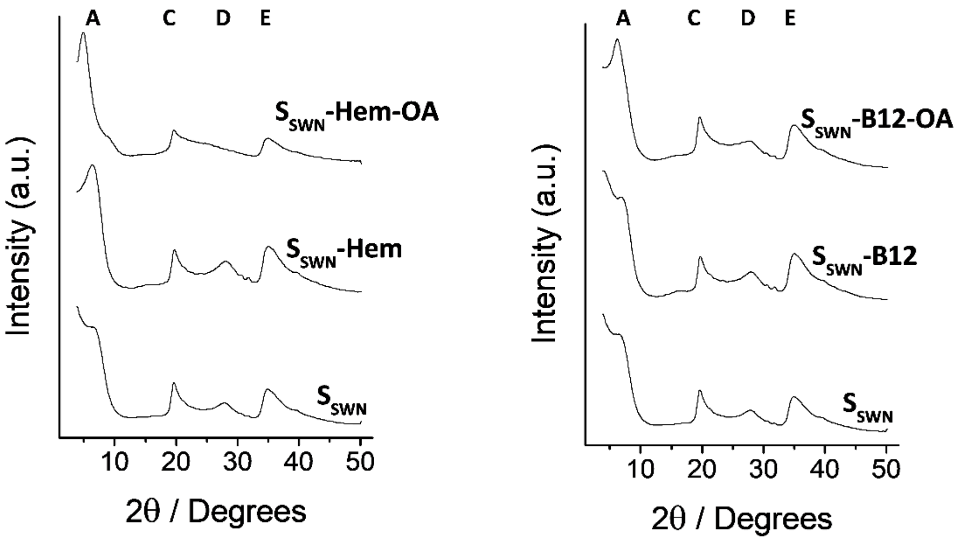

Figure S1. Normalized X-ray patterns of the bioactive organonanoclays (indicated in their respective diffractogram). From bottom to top: bare, loaded and final gated-organonanoclay, respectively. The main XRD reflections are labelled from left to right as A-E, consecutively. B-peak is omitted in order to correlate the peaks with the previously obtained for model-solids (Figure 1)

Table S1. Main reflexions' d-Spacing (Å) values for the bioactive organonanoclays obtained from Bragg's law ($n\lambda = 2d \cdot \sin(\theta)$, $\lambda = CuK_{\alpha\ av}$ = 1,54 Å, n=1).

	A	*B*	C	D	E
S_{SWN}	13.55		4.52	3.21	2.57
S_{SWN}-Hem	14.52		4.52	3.17	2.55
S_{SWN}-Hem-OA	18.03		4.53		2.54
S_{SWN}-B12	14.65		4.51	3.20	2.57
S_{SWN}-B12-OA	14.24		4.53	3.21	2.56

Loading and functionalization processes which lead to final gated-solids were followed by zeta (ζ) potential. The obtained values are reported in Figure S2, and they were measured in EtOH at 20 °C. As it can be observed, the bare S_{SWN} clay exhibited a negative ζ potential value (around -20 mV). After the loading process, the charge of the materials remains negative. Once the APTES molecule was added, ζ potential values become less negative, confirming the efficiency of the functionalization process, being the charge-neutralization a direct consequence of the presence of the APTES-amine group on the surface. Finally, the anchoring of OA to the particles increased the positivity in the ζ potential values of the particles.

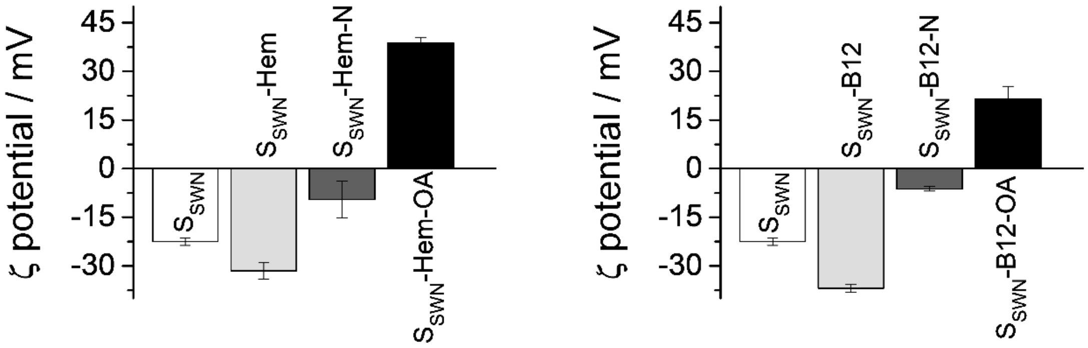

Figure S2. Zeta (ζ) potential values of all the bioactive organonanoclays. The increasing darkness in bar colors correspond to the progressive synthesis steps: from bare S_{SWN} to loaded, APTES-functionalized and final OA-functionalized organonanoclays (white, light gray, dark gray and black bars, respectively)

The bioactive organonanoclays in the different synthesis steps were also characterized by infrared spectroscopy (FTIR), and the obtained spectra are depicted in Figure S3. The dominant bands in all the FTIR spectra are those which belong to the silica tetrahedra and metal octahedra which conform the laminar structure of all the phyllosilicates (1050, 600 and 450 cm^{-1}). The broad band that appears around 3500 cm^{-1} is assigned to the vibration of the hydration water molecules. The hydroxyl group coordinated to octahedral cations, typically Mg^{2+}, show a small and sharp band at 3600 cm^{-1} and another sharp one at 1600 cm^{-1}. The clear appearance of two bands at approximately 2900 cm^{-1} and 2850 cm^{-1} in all the spectra of the gated clays is assignable to the bending C–H vibrations. These new bands are related with the presence of organic matter from the APTES or OA [4].

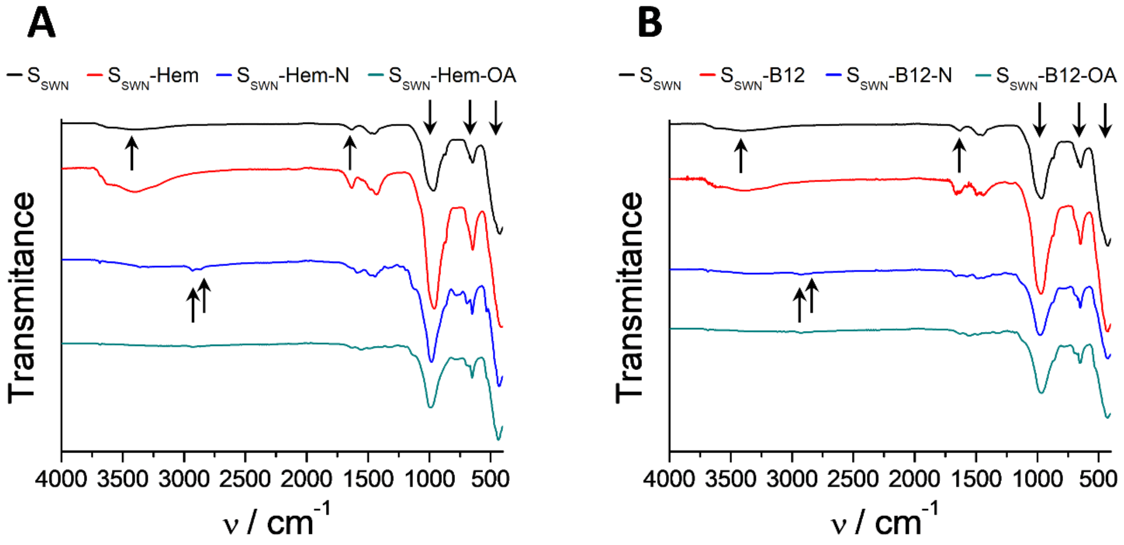

Figure S3. FTIR spectra of the consecutive synthesis steps of the bioactive organonanoclays: **A**, S_{SWN}-Hem-OA; **B**, S_{SWN}-B12-OA

Table S2. Parameters and coefficients of determination (r^2) obtained by adjusting the data with the K-P model (equation [3]) fixing the value of the *n* parameter (n ≥ 0.45).

System	K-P			
	K (min^{-n})	n	b	r^2
K_F-RhB-OA	5.65	0.45	1.87	0.98
S_{SA}-RhB-OA	6.53	0.45	5.04	0.96
S_{SWN}-RhB-OA	6.37	0.45	0	0.97
S_{SWN}-Hem-OA	0.01	1.21	0.71	0.96
S_{SWN}-B12-OA	4.52	0.45	32.97	0.72

RhB dye release from all the gated organonanoclays in gastric pH condition.

To obtain the cargo release profiles of all the model organonanoclays, 5 mg of the corresponding solids were placed in 10 mL of water (pH 2.5) simulating gastric conditions and 5 mg of Kf-Rh-OA 10 mL of PBS (pH 7.5) acting as a blank. At certain times (0.02, 0.25, 0.5, 1, 2, 4, 6, 8, 24 hours) aliquots of 700 µL were taken and filtered through 0.45 µm PTFE fil-ters. Finally, the RhB content was determined by fluorescence spectroscopy. For the meas-urement, the employed excitation and emission wavelengths were 555 and 572 nm, re-spectively. See Figure S4.

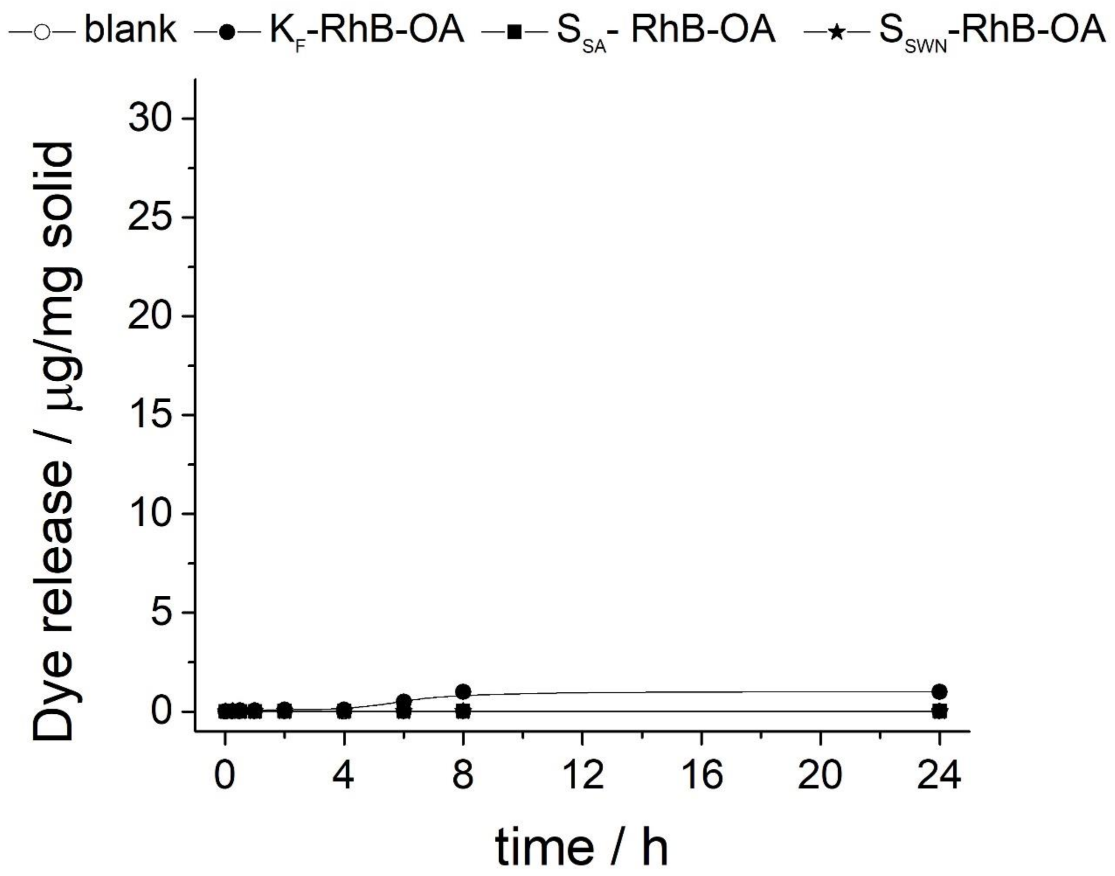

Figure S4. Release profile of RhB dye at pH 2.5 (○ blank, ● K_F-RhB-OA, ■ S_{SA}-RhB-OA, ★ S_{SWN}-RhB-OA)

RhB dye release experiment of smectite clays without OA coating

To obtain the cargo release profiles of all the model organonanoclays without OA coating, 5 mg of the corresponding solids were placed in 10 mL of PBS (pH 7.5). At certain times (0.02, 0.25, 0.5, 1, 2, 4, 6, 8, 24 hours) aliquots of 700 µL were taken and filtered through 0.45 µm PTFE filters. Finally, the RhB content was determined by fluorescence spectroscopy. For the measurement, the employed excitation and emission wavelengths were 555 and 572 nm, respectively. See Figure S5.

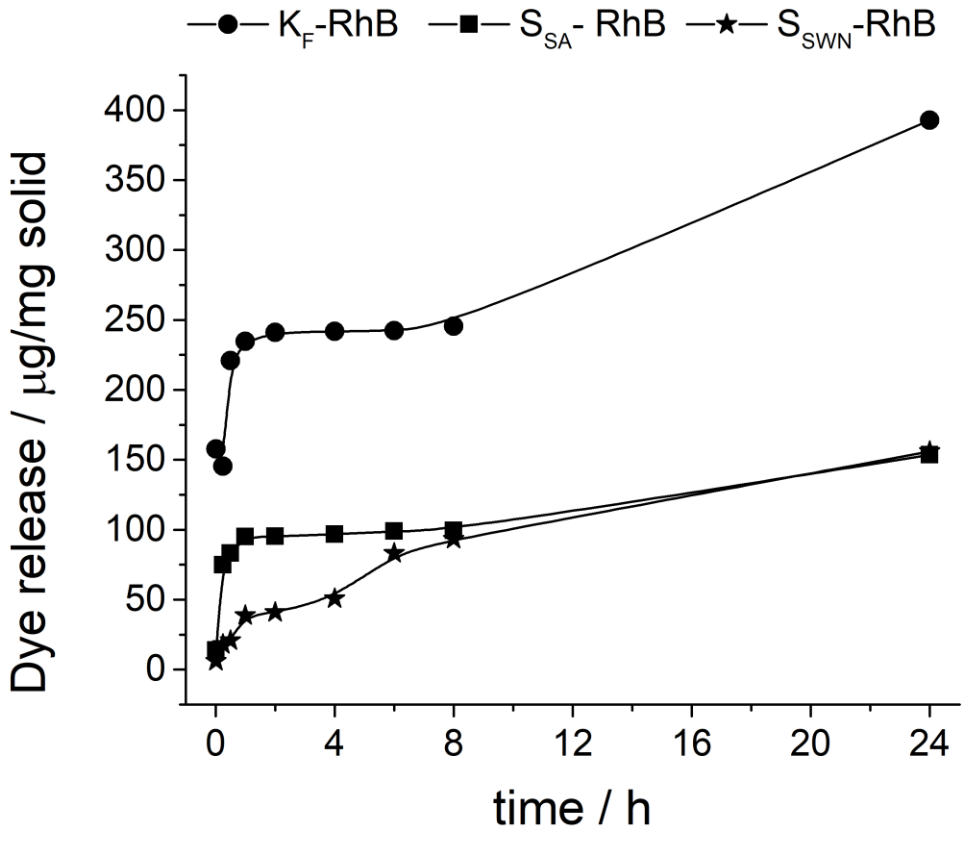

Figure S5. Release profile of RhB dye of smectite clays without OA coating at pH 7.5 (● K_F-RhB, ■ S_{SA}-RhB, ★ S_{SWN}-RhB)

Bioactive organonanoclays cargo delivery without OA coating

To obtain the cargo delivery profiles of the bioactive molecules without OA, 5 mg of the corresponding solid were placed in 10 mL of PBS. At certain times (0.02, 0.25, 0.5, 1, 2, 4, 6, 8, 24 h) aliquots of 700 µL were centrifuged and the bioactive molecule content determined by means of its absorbance in a UV-Vis spectrophotometer. For the measurement, the employed excitation wavelengths for *Hem* and *B12* were 385 and 361 nm, respectively. See Figure S6 and S7.

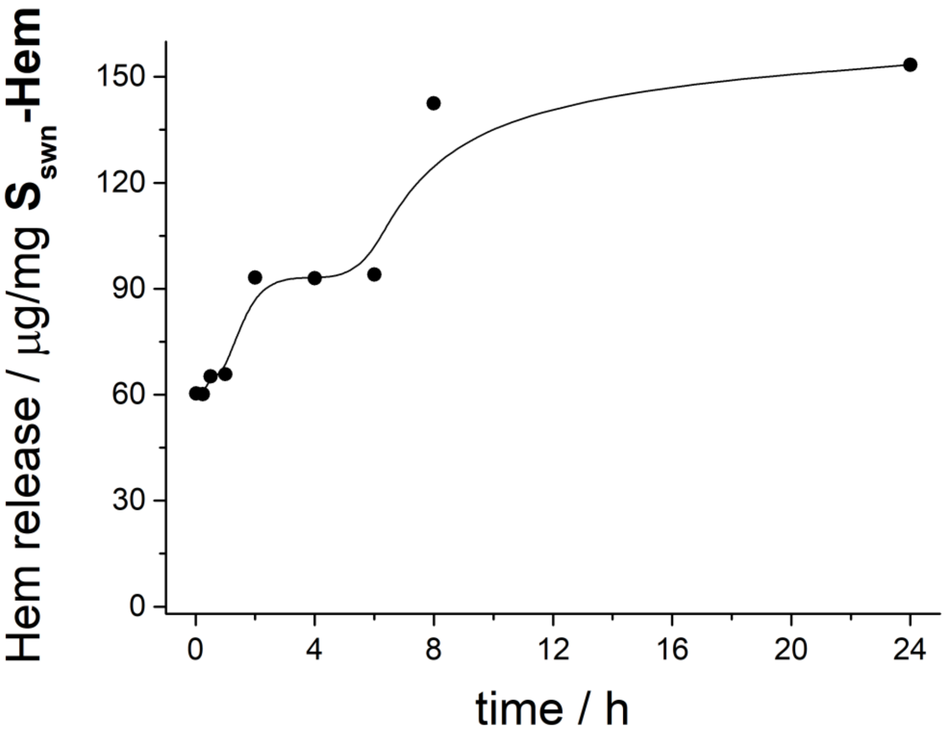

Figure S6. Release profiles of *Hem* at pH 7.5 from S_{SWN}-Hem.

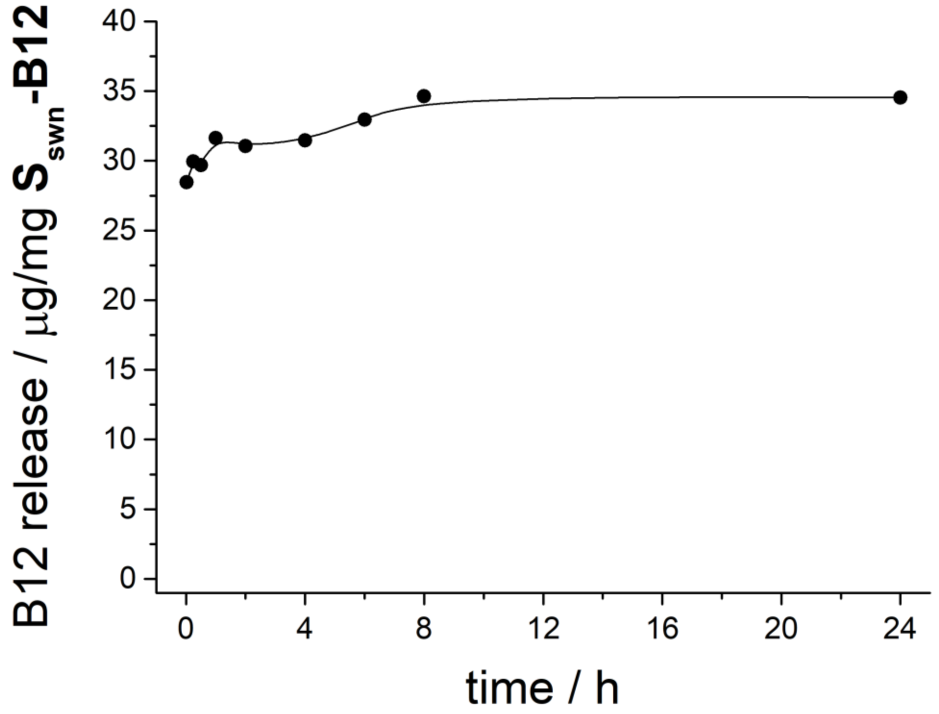

Figure S7. Release profiles of *B12* at pH 7.5 from, S_{SWN}-B12
